# Supplementary material for: Tantalum Nitride-Based Theranostic Agent for Photoacoustic Imaging-Guided Photothermal Therapy in the Second NIR Window
Source: Nanomaterials (Basel). 2023 May 23;13(11):1708. doi: 10.3390/nano13111708 (PMC10254667; doi:10.3390/nano13111708)
Supplement: Supplementary file 1 [file nanomaterials-13-01708-s001.zip › nanomaterials-2375023-supplementary.pdf]

Tantalum nitride-based theranostics agent for photoacoustic imaging-guided photothermal therapy in the second NIR window

Huixi Yi†, Gaoyang Yan†, Jinzhen He, Jiani Zhuang, Chengzhi Jin*, Dong-Yang Zhang*

Guangzhou Municipal and Guangdong Provincial Key Laboratory of Molecular Target & Clinical Pharmacology, the NMPA and State Key Laboratory of Respiratory Disease, the Second Affiliated Hospital and School of Pharmaceutical Sciences, Guangzhou Medical University, Guangzhou 511436, China

†Huixi Yi and Gaoyang Yan contributed equally to this work.

*Correspondence email: chengzhijin@gzhmu.edu.cn; zhangdy7@gzhmu.edu.cn.

Supporting Figures

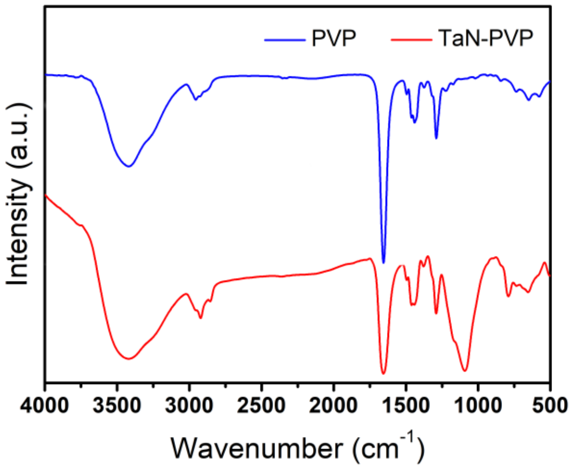

Figure S1. The FTIR spectra of PVP and TaN-PVP from 500 to 4000 cm^{-1}.

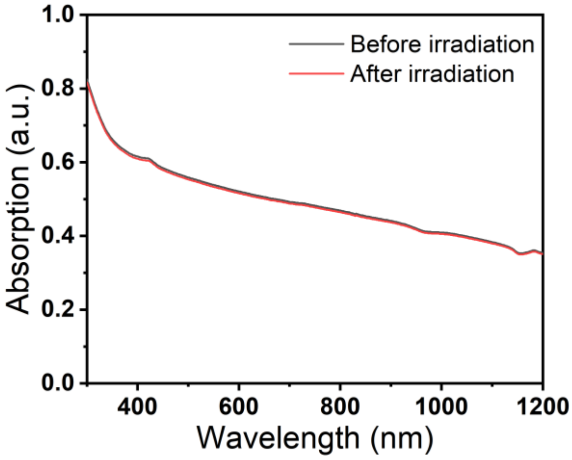

Figure S2. The absorption spectra of TaN-PVP NPs before and after irradiation with a 1064 nm laser for 20 min.

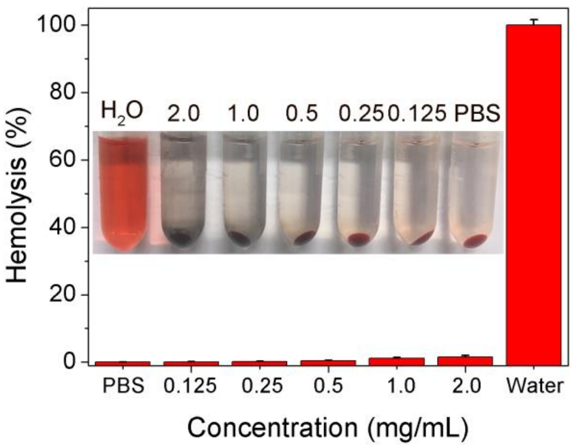

Figure S3. Hemolytic test of TaN-PVP NPs at indicated concentrations.

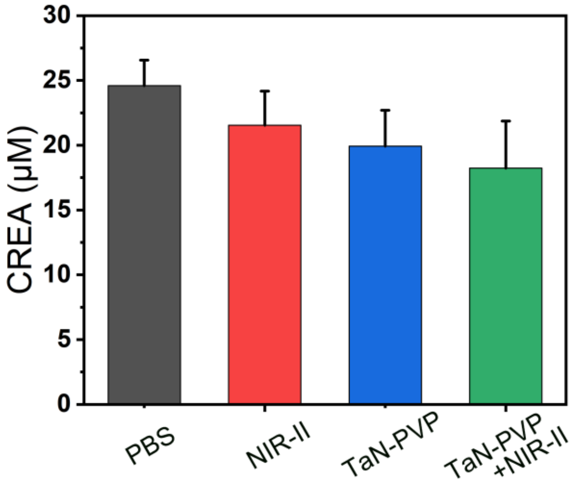

Figure S4. The level of CREA in serum from indicated groups.

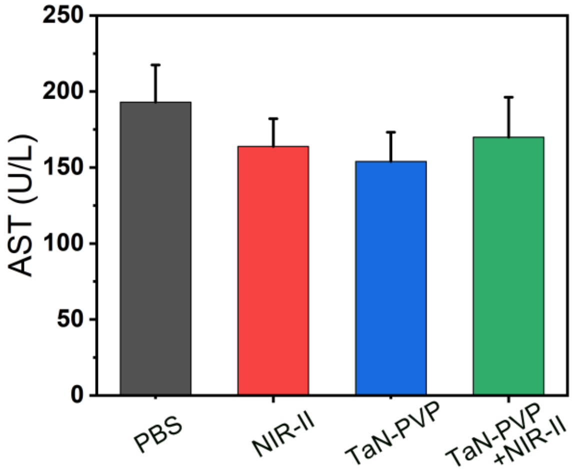

Figure S5. The level of AST in serum from indicated groups.
